# Cryoablation versus rib plating: Is the real problem pain control or chest wall instability?

**DOI:** 10.1016/j.tcr.2023.100858

**Published:** 2023-05-29

**Authors:** Jaron H. Butterfield, Laura B. Reparaz, Christopher M. Watson

**Affiliations:** Prisma Health Midlands, Trauma Critical Care Surgery, 9 Medical Park Drive, Suite 470, Columbia, SC 290203, United States of America

**Keywords:** Rib fixation, Intercostal nerve, Cryoablation, Rib fractures, Surgical stabilization rib fractures

## Introduction

A growing body of literature suggests improved outcomes in patients with rib fractures who undergo surgical stabilization of rib fractures (SSRF) [1]. As interest in rib fixation has grown, adjuncts to rib fixation and alternative techniques have developed to further improve pain control and minimize the morbidity of surgery [2]. An increasingly common reported adjunct is simultaneous intercostal nerve cryoablation (INCA) for durable pain control [3]. However, the contribution of INCA to overall outcomes remains unclear and its use alone as an adjunct therapy for rib fractures, apart from SSRF, has yet to be thoroughly studied to clarify its role and significance, or quantify the degree of benefit. Retrospective studies have suggested improved pain and opioid requirements when INCA is added to SSRF [4,5]. However, it is unknown from prospective randomized studies, if INCA with SSRF, decreases the need for narcotic pain medications in rib fractures and/or subsequent pulmonary complications, as compared to SSRF alone. Similarly, there are no prospective randomized studies to date evaluating the significance of INCA alone without SSRF. However, subjective improvement in pain control with INCA and SSRF has raised the question of whether the majority of the benefit is due to pain treatment or chest stabilization. Here, we present a case of a patient who sustained multiple bilateral rib fractures, and underwent SSRF with INCA on one side, but INCA alone for the contralateral side.

## Case presentation

**Fig. 1:**
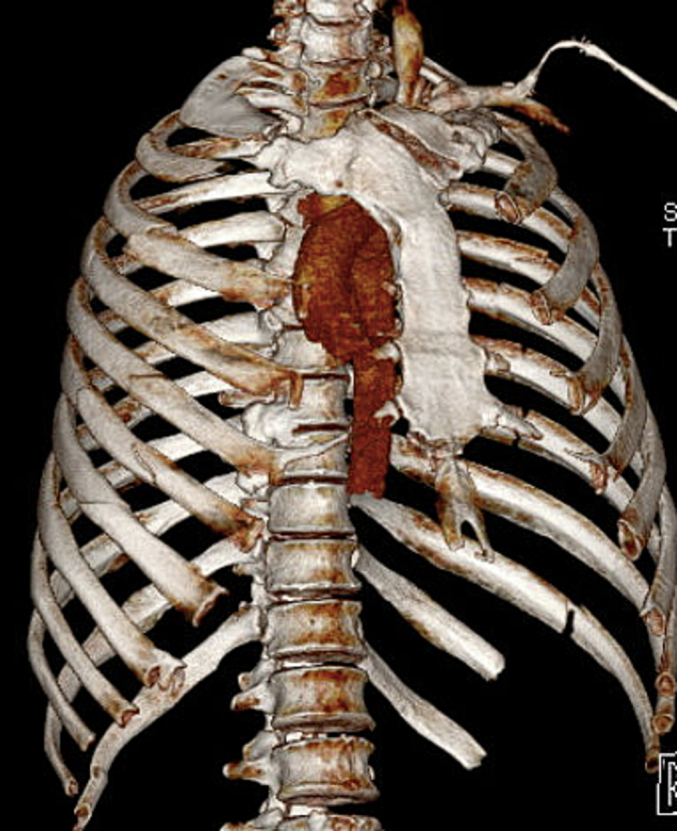
Preoperative CT chest with 3D Reconstruction demonstrating right-sided rib fractures.

**Fig. 2:**
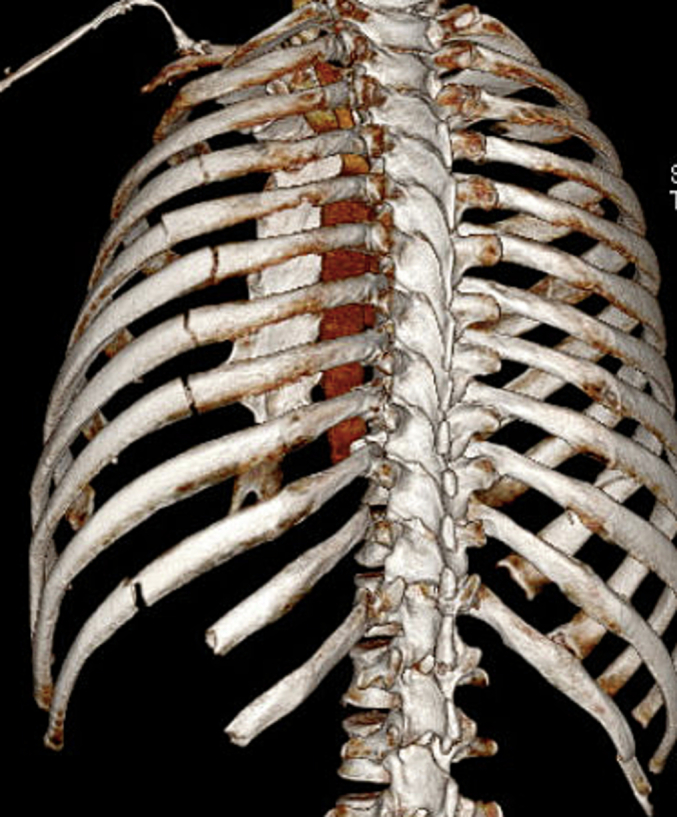
Preoperative CT chest with 3D Reconstruction demonstrating left-sided rib fractures.

A 56-year-old male presented to our trauma center after being involved in a motor vehicle collision with ejection. He was found to have some minor facial fractures and severe chest wall injuries including fractures of left ribs 4 through 8, and 10 through 12, fractures of right-sided ribs 2 through 7, right shoulder dislocation, right sided hemothorax, and fractures of the sternum and left clavicle. On presentation, the patient had significant respiratory distress with hypoxia, diminished forced vital capacity of 700 ml, and was admitted to the intensive care unit with high-flow nasal cannula therapy. He reported diffuse chest wall tenderness and subjective dyspnea. Multi-modal pain control was initiated with acetaminophen, gabapentin, a hydromorphone PCA, and a ketamine infusion. The sternal, clavicular, and shoulder dislocation injuries were managed non-operatively. The patient was additionally found to have blunt cardiac injury which appeared to improve with conservative management alone. Due to the number of bi-cortically fractured ribs bilaterally with displacement, and pulmonary physiologic derangements including diminished incentive spirometry, persistent uncontrolled pain, and poor cough, SSRF with INCA was recommended for chest wall stabilization. Prior to surgical intervention, the patient developed acute respiratory failure requiring intubation. The patient continued to experience hypoxia after intubation for several days, precluding internal thoracic plating. Bilateral fixation with external rib plating was planned. A CT of the chest with 3D reconstruction was used to plan operative approach and incision over the fracture line (Fig. 1, Fig. 2). This imaging demonstrated that several of the left sided lateral ribs had greater displacement than the minimally displaced bi-cortical right rib fractures.

**Fig. 3:**
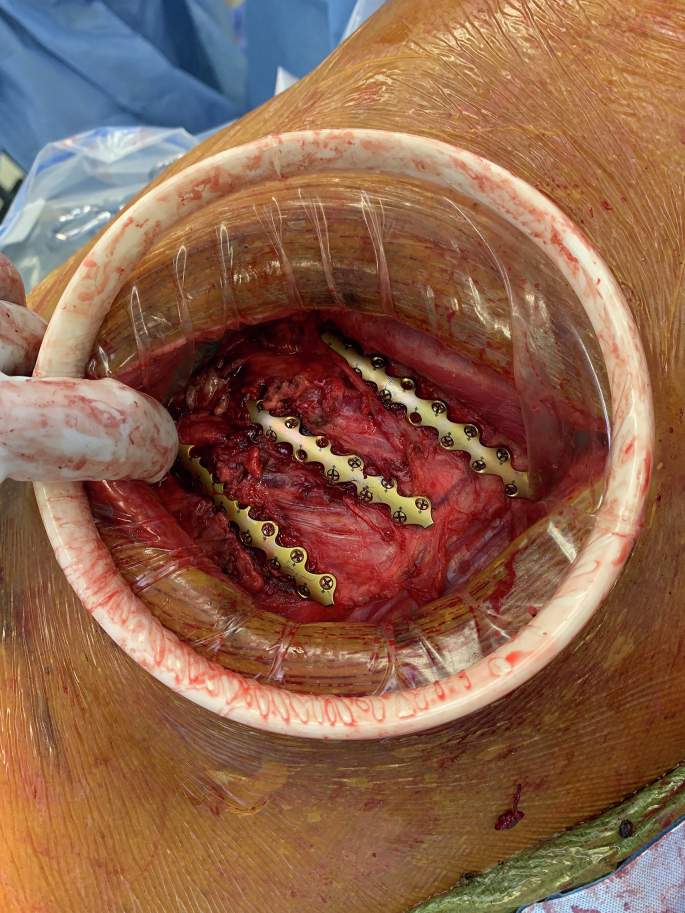
Intra-operative view of left-sided ribs after stabilization.

**Fig. 4:**
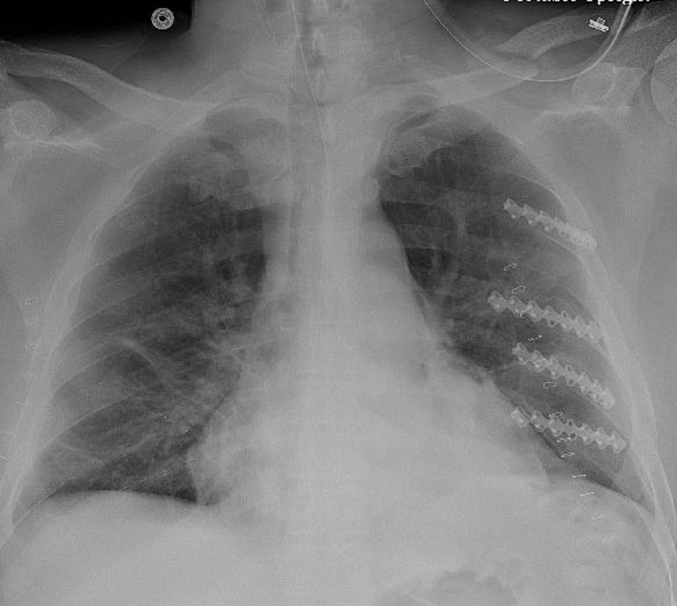
Postoperative chest X-ray demonstrating final hardware and bilateral lung expansion after chest tube removal.

On the day of surgery, the patient was brought to the operating room and placed in the right lateral decubitus position on a bean bag, and prepped and draped in the standard fashion. A 10 cm incision was made over the area of fractured ribs and continued to the latissimus muscle. The muscle was then split parallel to its fibers and retracted with an Alexis retractor to expose the chest wall. Displaced fractures of ribs 4, 6, 7, and 8 were confirmed. The ribs were reduced manually with reduction forceps. We then used a template to aid with plate contour and bending. A 16-hole external rib plate was then bent to shape to match the contour of the rib and fixed in place using multiple 7 mm locking screws on either side of the fracture line. This was performed for left-sided ribs 4, 6, 7, and 8 (Fig. 3). Cryoablation was then performed for intercostal nerves 3 through 8. A chest tube was placed through a separate incision and the surgical incision closed in layers. The patient was then returned to the supine position and then placed in the left lateral decubitus position and prepped and draped again. A similar 10 cm incision was made over the fracture line with similar dissection down to the chest wall. At this point, due to issues with patient ventilation and minimal fracture displacement identified, we elected to perform cryoablation alone for the right side, defer further rib plating and conclude the case. Cryoablation was then performed for right-sided intercostal nerves 3 through 8 and the skin was closed in layers. Bronchoscopy was performed after the procedure to aspirate all secretions. The patient was then able to safely extubate the following day and was downgraded from the ICU the day following. By post-operative day (POD) 4, the patient reported resolution of all chest pain and respiratory distress. Chest tubes were removed uneventfully with reassuring x-ray imaging (Fig. 4). The patient was evaluated one month after his accident and continued to deny any significant chest pain on either side.

## Discussion

Rib fractures and chest wall injuries have been clearly shown to be associated with compromised respiratory mechanics, which place patients at risk for subsequent hypoventilation and pneumonia. This has well-documented associated morbidity and mortality [6,7]. To improve the management of these injuries, a plethora of literature in the past decade has emerged to suggest various treatment options with various improved outcomes. Given that surgical management options and pain control therapies have evolved together, there remains an absence of robust literature to clarify the degree of contribution of each treatment. Although our patient had bilateral chest wall injury with bi-cortically fractured ribs on both sides, he was found at the time of surgery to only have significant displacement of fractures on his left side. Due to being intubated shortly after presentation, it was clinically difficult to assess whether one side had greater contribution to his respiratory failure. Regardless of difference in the degree of displacement, his rapid improvement after bilateral INCA but unilateral SSRF raises the question of whether or not his major issue was pain-related rather than due to chest wall instability. From a physiologic perspective, it is easy to understand how both pain and chest wall instability may be independent but interrelated factors contributing to symptoms and complications in chest wall injury. However, as mentioned already, their relationship is not well-quantified and clear from the current literature. A recent modified Delphi consensus among Chest Wall Injury Society members, identified INCA, among other topics, as a priority research interest in chest wall injury patients [8]. It is likely that prospective randomized studies to evaluate INCA alone, with and without SSRF, will continue to define INCA's impact on chest wall injury outcomes.

## Declaration of competing interest

The authors declare that they have no known competing financial interests or personal relationships that could have appeared to influence the work reported in this paper.
